# Plasma Metabolomic Profiling to Reveal Antipyretic Mechanism of *Shuang-Huang-Lian* Injection on Yeast-Induced Pyrexia Rats

**DOI:** 10.1371/journal.pone.0100017

**Published:** 2014-06-18

**Authors:** Xiaoyan Gao, Mingxing Guo, Qiang Li, Long Peng, Haiyu Liu, Li Zhang, Xu Bai, Yingxin Wang, Jian Li, Chengke Cai

**Affiliations:** 1 Science experiment center for TCM, Beijing University of Chinese Medicine, Beijing, China; 2 School of Chinese material medica, Beijing University of Chinese Medicine, Beijing, China; 3 Waters technologies (Shanghai) Ltd., Shanghai, China; 4 The 2^nd^ Traditional Chinese Medicine factory of Harbin pharm group CO. LTD, Harbin, China; 5 School of Basic Medical Sciences, Beijing University of Chinese Medicine, Beijing, China; National Research Council of Italy, Italy

## Abstract

*Shuang-huang-lian* injection (*SHLI*) is a famous Chinese patent medicine, which has been wildly used in clinic for the treatment of acute respiratory tract infection, pneumonia, influenza, etc. The existing randomized controlled trial (RCT) studies suggested that *SHLI* could afford a certain anti-febrile action. However, seldom does research concern the pharmacological mechanisms of *SHLI*. In the current study, we explored plasma metabolomic profiling technique and selected potential metabolic markers to reveal the antipyretic mechanism of *SHLI* on yeast-induced pyrexia rat model using UPLC-Q-TOF/MS coupled with multivariate statistical analysis and pattern recognition techniques. We discovered a significant perturbance of metabolic profile in the plasma of fever rats and obvious reversion in *SHLI*-administered rats. Eight potential biomarkers, i.e. 1) 3-hydeoxybutyric acid, 2) leucine, 3) 16∶0 LPC, 4) allocholic acid, 5) vitamin B_2_, 6) Cys-Lys-His, 7) 18∶2 LPC, and 8) 3-hydroxychola-7, 22-dien-24-oic acid, were screened out by OPLS-DA approach. Five potential perturbed metabolic pathways, i.e. 1) valine, leucine, and isoleucine biosynthesis, 2) glycerophospholipid metabolism, 3) ketone bodies synthesis and degradation, 4) bile acid biosynthesis, and 5) riboflavin metabolism, were revealed to relate to the antipyretic mechanisms of *SHLI*. Overall, we investigated antipyretic mechanisms of *SHLI* at metabolomic level for the first time, and the obtained results highlights the necessity of adopting metabolomics as a reliable tool for understanding the holism and synergism of Chinese patent drug.

## Introduction

Fever, also called pyrexia, was defined as a state of elevated core temperature, which is often, but not necessary, a part of the defensive responses of organisms (host) to the invasion of micro-organisms or inanimate matter recognized as pathogenic or alien [1]. It is also a cornerstone diagnostic sign in clinical practice that helps to carry out appropriate therapy in the early stage. Even though the febrile response seems useful in the adaptive reaction to a stressful situation, it could induce several detrimental effects on clinical outcomes, e.g. metabolic disorder, neurological injury [2], myocardial injury [3], pulmonary injury [4], and even worsens pre-existing disease. In general, febrile patients are frequently treated with kinds of antipyretic methods, including direct cooling, non-steroidal anti-inflammatory drugs (NSAIDs), and paracetamol, etc. Despite a lack of experimental and clinical data, these treatments may cause certain undesirable side effects, such as bleeding, hypotension, hepatic and renal injury [5]. Hence, it is complex for physicians and/or researchers to balance the benefits and harms of various therapeutic means.

Over the past thousands of years, traditional Chinese medicine (TCM) has been used to treat fever by practitioners according to their experience and heritage in China. Nowadays, TCM is attracting considerable attentions worldwide due to its low toxicity yet effective therapeutic performance [6]. In recent years, some modern formulae (e.g. *Qing-kai-ling* injection [7], [8], *Nao-re-qing* oral liquid [9], *Tan-re-qing* injection [10], [11], etc.) showed great anti-febrile action in clinic. Among them, *Shuang-huang-lian* injection *(SHLI),* which derived from three herbal drugs (*Lonicera Japonica*, *Scutellariae Radix,* and *Forsythiae Fructus*), is a commonly used TCM preparation with officially recorded in the Chinese Pharmacopoeia (2005). Although considerable numbers of chemical compounds are contained, three effective ingredients, *caffeotannic acid, baicalin and phillyroside*, are officially recorded as quality control standards. Since 1973, SHLI has been used extensively in clinic to treat infectious diseases, such as pneumonia, influenza, acute tonsillitis, and acute faucitis [12], [13]. The existing clinical randomized controlled trials (RCT) suggest that all *SHL* preparations (tablet, oral liquid, injection, and so on) could show certain anti-febrile action (in Chinese) [14], [15]. However, there are seldom researches focusing on the pharmacological mechanisms of antipyretic effect of *SHL* preparations, and even most of the results were extracted from some routine tests on conventional animal models, such as resisting free radicals damage, raising immune response, inhibiting inflammatory factor (TNF-α, IL-1, IL-6, etc.), and influencing PGE2 related pathways [8], [16]. Therefore, it is necessary to introduce in-depth study for the antipyretic effect of *SHLI* using some holistic techniques owing to the ill-defined underlying mechanisms of this famous formula.

In comparison with conventional research tools, metabolomics could address some novel features for studying the pathophysiological characteristics and pathogenesis of diseases by comprehensively monitoring low-molecular-weight endogenous substances using some advanced data acquiring methods, such as LC-MS, NMR, GC, and so on [17]–[20]. Among these techniques, ultra performance liquid chromatography coupled with quadrupole time-of-flight mass spectrometry (UPLC-Q-TOF/MS) has widely used for metabolomic analysis [21], [22]. Owing that UPLC possesses great peak capacity and chromatographic resolution due to its special column with stationary phase of 1.8 µm particles and Q-TOF/MS can provide the chromatographic peak area, accurate mass value of *m/z*, and abundant structural information for the eluate from LC domain.

In this study, pyrexia rat model was developed by injecting aqueous suspension of yeast and plasma samples were collected from normal, model and *SHLI*-treated model groups, while UPLC-Q-TOF/MS was introduced for the metabolite analysis. At the meanwhile, multivariate statistical analysis in combination with pattern recognition techniques and metabolic pathway analysis were adopted to gain potential plasma markers for yeast-induced pyrexia. Moreover, *SHLI* was administrated to the model animals in parallel aiming to gain mechanistic insights for their antipyretic effects.

## Results

### Temperature Changes Measurement Results

**Figure 1:**
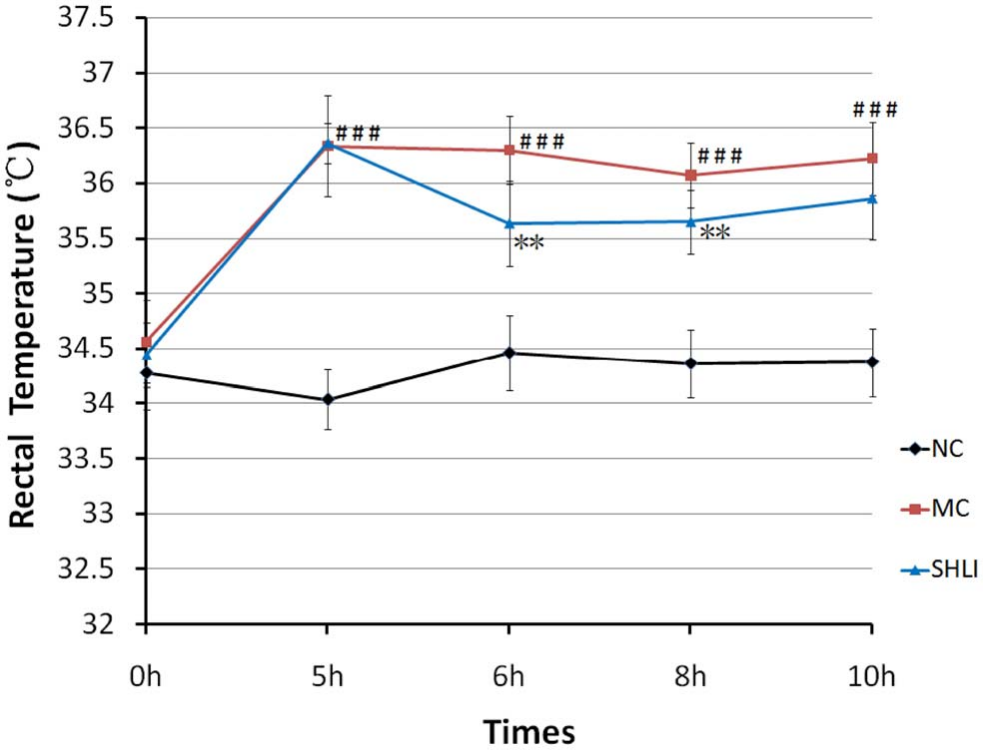
The changing trend of rectal temperatures in NC, M and *SHLI* treatment group. Data was expressed as mean ± SD. ^###^ *P*<0.001 vs., NC group; **P<0.01 vs., M group.

To investigate the antifebrile action of *SHLI*, the rectal temperatures of the rats at different time points were recorded (Fig. 1). The results showed that body temperature significantly increased 5 hrs after yeast injecting (*p<0.01 vs*. node control group). On the other side, *SHLI* could markedly decrease the body temperature at the checking points of 6 hr and 8 hr (*p<0.01 vs*. model group), suggesting a definite antifebrile action for *SHLI*. As a consequence, the time point of 6 hr was selected for subsequent metabolomic analysis.

### Multivariate Data Analysis of UPLC-ESI-Q-TOF/MS Data

**Figure 2:**
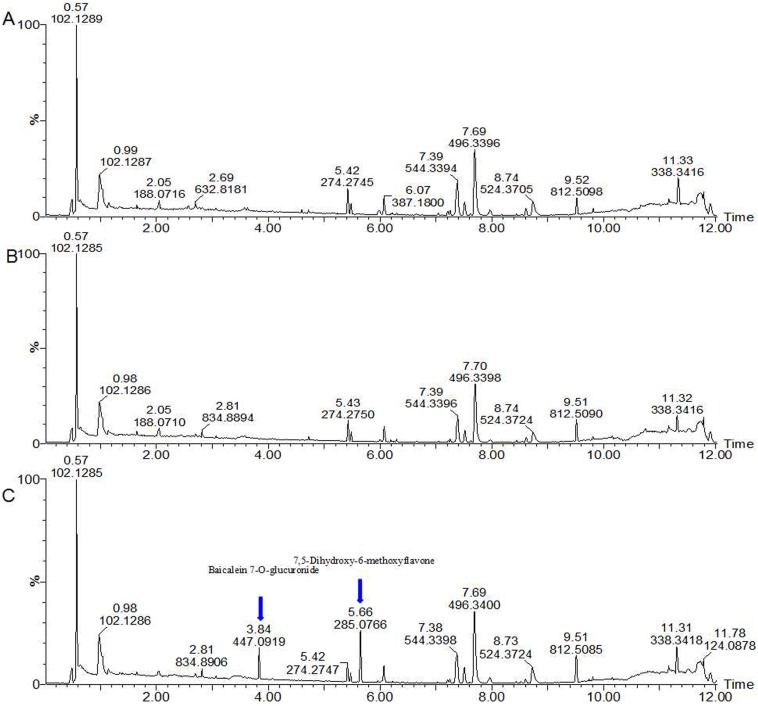
Typical base peak intensity (BPI) chromatograms ofplasma samples from each groups. (A) NC at positive ion mode. (B) M at positive ion mode (C) *SHLI* treatment at positive mode (Blue arrows show drug induced components).

**Figure 3:**
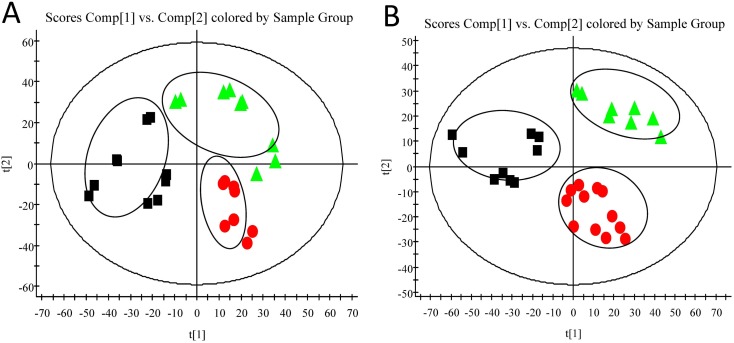
The results of multiple pattern recognition of plasma metabolites (PCA scores). (A) At positive ion mode. (B) At negative ion mode. Note: NC (▴), M (•) and SHLI (▪).

Typical UPLC-Q-TOF/MS base peak intensity (BPI) chromatograms of plasma samples from the normal control (NC), model (M) and *SHLI*-treated groups were illustrated in Fig. 2. Some obvious drug-related components could be observed in Fig. 2C (marked with blue arrows). As well known, drug-related components in plasma might interfere with the profile of unsupervised multivariate data analysis. In order to overcome the interference, the drug-related components with high contents in plasma were identified and deducted (See Table S1). An unsupervised principle component analysis (PCA) method was used to intuitively characterize the disturbance among NC, M and *SHLI*-treated group (Fig. 3A and 3B). As shown in Fig. 3 (R^2^X = 0.744, Q^2^ = 0.444 in positive ionization mode; R^2^X = 0.543, Q^2^ = 0.327 in negative ionization mode), obvious separation was achieved with the stable cumulative modeled variation and good prediction capability by the first two principle components under two kinds of ionization modes. The results suggested that the perturbation of plasma metabolic profile occurred in rats after yeast administrated and *SHLI* treated.

### Identification of Metabolites

**Figure 4:**
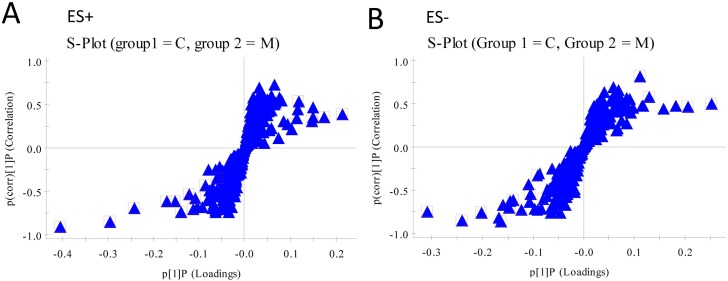
The results of S-plots of OPLS-DA models. (A) At positive ion mode. (B) At negative ion mode. Note: NC (▴), M (•) and SHLI (▪).

**Figure 5:**
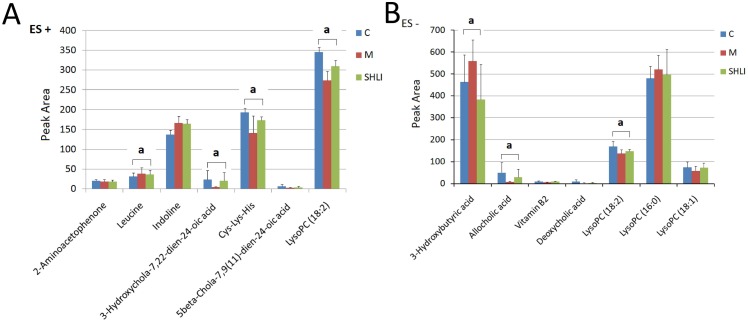
The results of relative integral levels of metabolites among NC, M and *SHLI* treatment groups. (A) At positive ion mode. (B) At negative ion mode. ^a^ *P*<0.05 or *P*<0.01 among NC, M, and *SHLI* treatment group.

A supervised orthogonal partial least squares discriminant analysis (OPLS-DA) technique was implemented to search distinct metabolites between M and NC groups. The parameter R^2^Y (0.966 in positive ion mode; 0.982 in negative ion mode) indicated that the established model was capable of differentiating NC group from M group. The parameter Q^2^ (0.885 in positive ion mode; 0.939 in negative ion mode) showed that the established model owned strong predictability. The OPLS-DA S-plots were shown in Fig. 4, and potential markers were extracted based on their contribution to the variations and correlation within the dataset. Based on VIP >1 and the perturbanced degrees of the metabolites evaluated by Student’s *t*-test and Mann-Whitney U test, 15 metabolites were identified as potential biomarkers and listed in Table 1. Among them, 5 metabolites in the M group, including Cys-Lys-His (*P*<0.001), allocholic acid (*P*<0.01), 3-hydroxychola-7,22-dien-24-oic acid (*P*<0.01), vitamine B2 (*P*<0.01), and LysoPC (18∶2) (*P*<0.001) decreased obviously comparing with the NC group, while 3 ones in the M group, including 3-hydroxybutyricacid (*P*<0.05), leucine (*P*<0.05), and LysoPC (16∶0) (*P*<0.05), increased significantly comparing with the NC group. Following SHLI treatment, the changes of 8 out of the 15 metabolites was significantly reversed (Fig. 5). All the results above suggested that *SHLI* treatment could effectively regulate some metabolic networks associated with their related metabolites in the yeast induced pyrexia rats.

**Table 1:** Metabolites selected by OPLS-DA with VIP >1 and significant test *P*<0.05 between the pyretic model group and the normal control group.

No.	t_R_-*m/z*	VIP	Quasi-molecular ion	Formula	Metabolites	M *vs.*C	SHLI *vs.*M	MS/MS	Loss	Related Pathway
**1**	1.2403–136.0763	1.58	[M+H]^+^	C_8_H_9_NO	2-Aminoacetophenone	↓^#^	↑	119.0490; 91.0678	−NH_3_; −C_2_H_3_O	Unknown
**2**	1.2996–132.1027	1.09	[M+H]^+^	C_6_H_13_NO_2_	Leucine^a^	↑^#^	↓*	86.0968; 69.0701; 55.0398	−CH_2_O_2_; −CH_5_O_2_N; −C_2_H_7_O_2_N	Valine, leucine and isoleucine degradation/biosynthesis; Glucosinolate biosynthesis; Aminoacyl-tRNA biosynthesi
**3**	1.6469–120.0815	1.91	[M+H]^+^	C_8_H_9_N	Indoline	↑^# #^	↓	104.0626	−NH_2_	Unknown
**4**	5.9690–373.2733	3.19	[M+H]^+^	C_24_H_36_O_3_	3-Hydroxychola-7, 22-dien-24-oic acid	↓^# #^	↑*	355.2595; 227.1801; 201.1626	−H_2_O; −C_7_H_14_O_3_; −C_9_H_16_O_3_	Secondary bile acid biosynthesis
**5**	6.0673–387.1802	4.41	[M+H]^+^	C_15_H_26_N_6_O_4_S	Cys-Lys-His	↓^# # #^	↑**	231.1104; 105.0709	−C_5_H_8_N_4_S; −C_12_H_18_O_2_N_4_S	Unknown
**6**	6.1132–357.2787	2.31	[M+H]^+^	C_24_H_36_O_2_	5beta-Chola-7, 9 (11) -dien-24-oic acid	↓^# #^	↑	339.2680; 247.1692; 111.1143	−H_2_O; −C_8_H_14_; −C_16_H_22_O_2_	Secondary bile acid biosynthesis
**7**	7.3612–520.3396	5.01	[M+H]^+^	C_26_H_50_NO_7_P	LysoPC (18∶2)	↓^# # #^	↑***	502.3279; 240.1013; 184.0727	−H_2_O; −C_18_H_32_O_2_; −C_21_H_36_O_3_	Glycerophospholipid metabolism
**8**	1.1853–103.0396	4.54	[M-H]^−^	C_4_H_8_O_3_	3-Hydroxybutyric acid (3-HB)^a^	↑^#^	↓**	85.0301; 59.0141	−H_2_O; −C_2_H_4_O	Synthesis and degradation of ketone bodies; Butanoate metabolism; Metabolic pathways
**9**	**3.6313–453.2850**	1.80	[M+FA-H]^−^	C_24_H_40_O_5_	Allocholic acid^a^	↓^# #^	↑*	407.2804; 343.2635; 289.2235; 251.2093	−CH_2_O_2_; −C_2_H_6_O_5_; −C_6_H_12_O_5_; −C_9_H_14_O_5_	Secondary bile acid biosynthesis
**10**	3.7077–421.1409	1.04	[M+FA-H]^−^	C_17_H_20_N_4_O_6_	Vitamin B2^a^	↓^# #^	↑***	255.0880; 241.0738; 212.0816	−C_5_H_10_O_6_; −C_6_H_12_O_6_; −C_12_H_10_N_4_O_3_	Riboflavin metabolism; Vitamin digestion and absorption
**11**	3.7717–448.3059	1.84	[M-H]^−^	C_26_H_43_NO_5_	Deoxycholic acid glycine conjugate	↓^# #^	↓	294.1783; 257.1782	−C_10_H_18_O; −C_9_H_19_O_4_	Secondary bile acid biosynthesis
**12**	4.3496–391.2858	1.31	[M-H]^−^	C_24_H_40_O_4_	Deoxycholic acid^a^	↓^# #^	↑	345.2807; 329.2882; 327.2711; 311.2400	−CH_2_O_2_; −CH_2_O_3_; −CH_4_O_3_; −CH_4_O_4_	Secondary bile acid biosynthesis
**13**	4.3993–564.3294	2.57	[M+FA-H]^−^	C_26_H_50_NO_7_P	LysoPC (18∶2)^a^	↓^#^	↑	504.3081; 279.2325; 224.0688	−C_2_H_5_O_2_; −C_9_H_21_O_7_NP; −C_20_H_43_O_5_	Glycerophospholipid metabolism
**14**	4.6068–540.3299	2.48	[M+FA-H]^−^	C_24_H_50_NO_7_P	LysoPC (16∶0)^a^	↑^#^	↓*	480.3089; 255.2323	−C_2_H_5_O_2_; −C_9_H_21_O_7_NP	Glycerophospholipid metabolism
**15**	4.7769–566.3451	1.43	[M+FA-H]−	C_26_H_52_NO_7_P	LysoPC (18∶1)	↓^#^	↑	506.3269; 281.2488	−C_2_H_5_O_2_; −C_9_H_21_O_7_NP	Glycerophospholipid metabolism

Note: ^a^Metabolites were identified based on database information in METLIN, Lipid MAPs or HMDB; ↑showed up-regulated metabolites and ↓showed down-regulated metabolites; ^#^ *p<0.05*, ^# #^ *p<0.01*, ^# # #^ *p<0.001* Model vs. normal control; *p<0.05, **p<0.01, ***p<0.001 SHLI vs. Model.

### Metabolic Pathway Analysis of Drug Target Candidates

**Figure 6:**
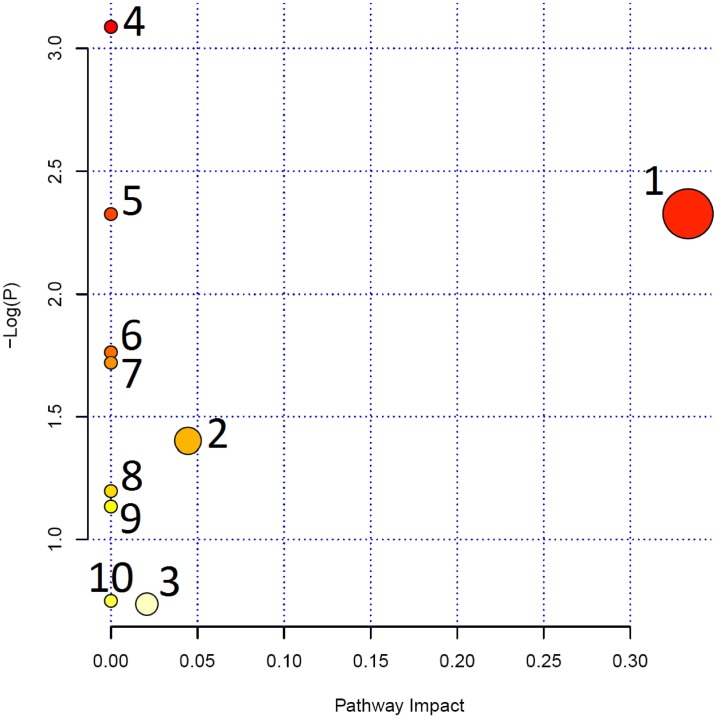
Summary of pathway analysis with MetPA. Note: 1. Valine, leucine and isoleucine biosynthesis. 2. Glycerophospholipid metabolism. 3. Synthesis and degradation of ketone bodies. 4. Riboflavin metabolism. 5. Butanoate metabolism. 6. Valine, leucine and isoleucine degradation. 7. Aminoacyl-tRNA biosynthesis.

To further reveal the correlation among the distinct candidates, bioinformatics analysis was performed using MetPA, a web-based free tool that could combine results from powerful pathway enrichment analysis with the pathway topology analysis. As shown in Fig. 6, the selected metabolites were involved in valine/leucine/isoleucine biosynthesis/degradation, glycerophospholipid metabolism, synthesis and degradation of ketone bodies, riboflavin metabolism, butanoate metabolism, secondary bile acid biosynthesis and aminoacyl-tRNA biosynthesis (Table S2), suggesting that the target pathways might be the diagnostic perturbations during pyrexia and might be the targets for the treatment of *SHLI*.

## Discussion

In this study, we firstly evaluated the antipyretic effect of *Shuang-huang-lian* injection (*SHLI*) using UPLC-Q-TOF/MS based metabolomic study. SHLI is a famous Chinese patent medicine and has been developed in a freeze-dried powder form by Harbin Pharmaceutical Group in 1990s. In recent years, *SHLI* has drawn widespread attention and the market demand increase dramatically owing to its wide distribution and potential biological effects. In 2009, *SHLI* was shortlisted by the ministry of health of China as one of the recommended treatments against influenza A H1N1 virus [23].

Although *SHLI* is one of the major drugs to treat pyrexia (fever) in clinic, there is still little information concerning its antipyretic mechanism [24]. As we known, fever is a type of nonspecific and phylogenetic response to various infectious or non-infectious stimuli. Yeast-induced fever (also called pathogenic fever), which is usually accompanied by certain radical and inflammatory reaction caused by capsular polysaccharides and/or proteins of yeast, is a classical model for pharmacological study of antipyretic medicines [25]. GC/MS combined NMR has been adopted to analyze the metabolic profiling in the plasma in fever rat induced by baker’s yeast and the antipyretic effect of aspirin was also evaluated by metabolomic methods, while those results indicated that the perturbation of amino acid metabolism coupled with energy metabolism, lipid metabolism, and glycometabolism were involved in the yeast induced fever model [26]. Moreover, we have investigated the metabolic profiling and potential biomarkers in the urine from yeast induced pyrexia rats using UPLC/MS technique, and our previous results were in great agreement with that amino acid metabolism played a pivotal role on the pathogenesis of fever [27]. Furthermore, we also studied the antipyretic effects of *Qing-kai-ling injection* using UPLC/MS based metabolomic study [7]. Honestly, pharmacological mechanisms of tradition Chinese preparation are unpredictable and poorly understood due to not only the complexity of multiple chemical ingredients, but also the multiple targeting sites involved during its *in vivo* process. Therefore, it is important to adopt comprehensive techniques to address this shortcoming.

At present study, we explored the plasma metabolic profiling and potential metabolic markers to investigate the antipyretic effects of *SHLI* using the well established research strategy [7], [27]. The results indicated that the metabolic alteration including increment of 3 metabolites in plasma (3-hydeoxybutyric acid, leucine, and 16∶0 LPC), and decrement of 5 metabolites in plasma (allocholic acid, vitamin B_2_, Cys-Lys-His, 18∶2 LPC, and 3-hydroxychola-7, 22-dien-24-oic acid) occurred after yeast treatment. It is noteworthy that all the changes of these metabolites could be reversed according to *SHLI* treatment, suggesting the possible pharmacological mechanisms of antipyretic effects of *SHLI*. In other words, *SHLI* might contribute to repair these metabolites involved pathways, i.e. lipid metabolism (glycerophospholipid metabolism, ketone bodies synthesis and degradation, bile acid biosynthesis), amino acid metabolism (valine, leucine and isoleucine biosynthesis/degradation) and energy metabolism. Regarding these, a published similar study arouses our intense interest. Guang-yan Yan et al. has adopted NMR-based metabolomic approaches to investigate the toxicological effects of SHLI in Beagle dog [28]. They showed that high-dose SHLI could lead to hemolytic anemia and hepatic hemosiderosis due to membrane impairment and hemolytic effects. And their metabolomic investigations suggested that the toxicological profiling of high-dose SHLI might link disordered energy and lipid metabolism processes [28]. Even more remarkably, most of the toxic related metabolic biomarkers of SHLI, e.g. 3-hydeoxybutyric acid, choline, phosphocholine, valine, and leucine, were also detected on the antipyretic action in SHLI treatment group. These findings highlight the pharmacological characteristics and safety evaluation of SHLI [29], [30]. Furthermore, the further experiments should be taken fully into account a dose and time dependent manners of SHLI. And the limitation of this study is that we only focused on the pharmacological mechanisms without the adverse drug reaction (ADR) profiles of SHLI.

**Figure 7:**
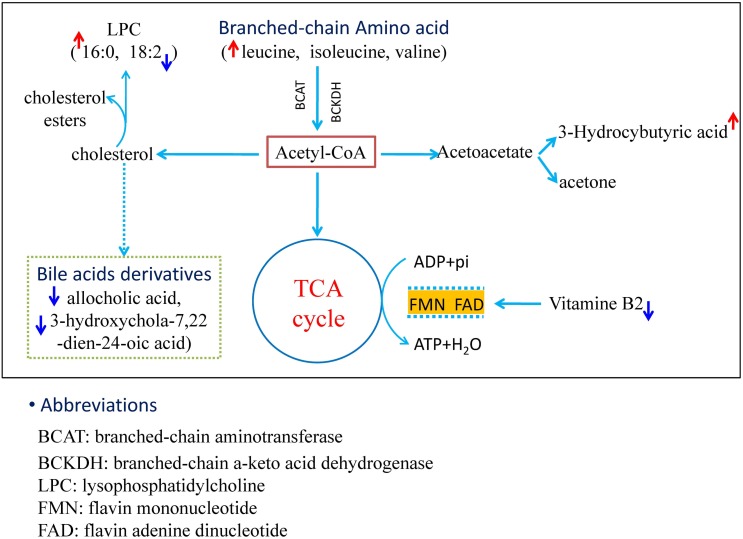
The profile of metabolic network. The map was gained by analyzing the known metabolic pathways.

According to the current knowledge of metabolic pathways, we mapped the relationship network of these metabolites. As shown in Fig. 7, the accelerated energy metabolism involving oxidation-reduction reactions, branched-chain amino acid metabolism, cholesterol metabolism, and ketone bodies metabolism were found to link with the significantly modified metabolites. Therefore, the antipyretic mechanism of *SHLI* was performed by correcting the perturbed metabolism of energy by a variety of metabolic pathways based on the results obtained from plasma metabolomic profiling.

3-Hydroxybutyric acid (3-HB), also called *β*-hydroxybutyric acid or *β*-hydroxybutyate, is a kind of carboxylic acid (ketone body) which synthesized in the liver from acetyl-CoA and can be used as an energy source by the brain when blood glucose is insufficient [31]–[34]. 3-Hydroxybutyric acid concentration increased in the plasma of the fever rats, indicating that fatty acid disintegration was enhanced to furnish energy. Leucine, a branched chain amino acid, is an essential amino acid which cannot be synthesized by animals. Since dietary leucine intake during the study period was likely zero (imposition of fasting), the increased leucine in plasma possibly stems from protein decomposition, indicating a negative nitrogen balance state in the modeling body. This result was in agreement with the similar researching reports [26]. It was reported that leucine metabolism could be affected in tumor necrosis factor α (TNF-α) and endotoxin (such as LPS) treated rats [35], suggesting that TNF-α or LPS can increase leucine oxidation and clearance of whole body which might subsequently affect the production of L-enkephalin in the hypothalamus. In addition, branched-chain amino acids that participate in valine/leucine/isoleucine biosynthesis and degradation might change the metabolic environment inside the blood-brain barrier [36].

LysoPC (lysophosphatidylcholine, LPCs), also called lysolecithins, is a class of phospholipids derived from phosphatidylcholines, which is formed by phosphatidylcholine hydrolysis by the enzyme of phospholipase A_2_, as a part of the de-acylation/re-acylation cycle that controls its overall molecular species composition. The other LPC formation pathway catalyzes by a specific enzyme system, i.e. lecithin-cholesterol acyltransferase (LCAT), which is an enzyme that could convert free cholesterol into cholesteryl ester. Since LPCs has cytolytic and membrane perturbing properties, the level of them must be strictly controlled. The increased level of LPCs in the plasma was considered as a marker for cell membrane injury. Researchers have found that LPC increases under reactive oxygen species (ROS) and inflammatory conditions, such as patients suffering from liver injury, lung infection, and diabetes, etc [37], [38]. Interestingly, the level of the saturated LPC (16∶0) increased, while the content of unsaturated LPC (18∶2) decreased in the plasma of fever rats. It was known that LPC species exhibited different capacities in the induction of cyclooxygenase-2 (COX-2) and prostaglandins (PGs) expression due to the length and the saturation degree of LPC acyl chains, i.e. 16∶0 LPC possessed more pronounced capacity than 18∶2 LPC [39]–[41]. Therefore, the increase of 16∶0 LPC could elevate PGE2 levels in the plasma of fever rats which could give rise to pyrexia finally. However, the relationships between plasma levels of LPCs and fever were not known, which calls for further evidences and much more explorations in-depth.

As an essential dietary, vitamin B2 (or riboflavin) is the main source of flavin mononucleotide (FMN) and flavin adenine dinucleotide (FAD), and is therefore required by all favoproteins. As we known, vitamin B2 plays a key role in energy metabolism, and for the metabolism of fats, ketone bodies, carbohydrates, and proteins [42]–[44]. Moreover, as coenzymes, FMN and FAD play a wide role on oxidation-reduction reactions in metabolic transformations by aiding one or two electrons [45]–[47].

Since the limitations of the analysis based on MetPA, some biomarkers such as allocholic acid and 3-hydroxychola-7, 22-dien-24-oic acid were not matched in the pathway analysis. In order to find the contribution of pyrexia, we searched them manually in documented data and biochemical databases. Allocholic acid and 3-hydroxychola-7, 22-dien-24-oic acid, both of which was in the plasma of pyrexia rats, belong to bile acids and their derivatives that are the metabolites of cholesterol. Previous reports have documented that neutropenic patients with fever showed a significant decrement for the level of serum cholesterol [48], indicating that the decrease bile acids in pyrexia rats might be related to the cholesterol decrease.

## Conclusion

In summary, we discovered a significant perturbance of metabolic profile in the plasma of yeast induced fever rats, which could be obviously reversed by *SHLI* treatment. The metabolic profiles alteration was characterized not only by the increase of plasma 3-hydeoxybutyric acid, leucine, 16∶0 LPC, but also by the decrease of plasma allocholic acid, vitamin B_2_, Cys-Lys-His, 18∶2 LPC, and 3-hydroxychola-7, 22-dien-24-oic acid following yeast administration. Based on metabolic pathway analysis and particular explanations, we concluded that the antipyretic mechanism of *SHLI* lay on correcting perturbed energy metabolism according to a variety of metabolic pathways. Taken together, the findings obtained in current study can be used to predict some possible targets for fever and *SHLI* treatment. Furthermore, our study also highlights the importance of metabolomics as a potential tool for uncovering metabolic pathways to discover targets for understanding the holism and synergism of Chinese medicine formulas.

## Materials and Methods

### Chemicals and Materials

Yeast was purchased from Mauri Food Co., Ltd. (Hebei, China). HPLC grade formic acid was obtained from Sigma Chemical Co., Ltd (St. Louis, MO, USA). Methanol and acetonitrile (HPLC grade) was acquired from Fisher Corporation (Michigan, USA). Ultra high purity water was prepared by Millipore-Q SAS 67120MOLS HEIM (France). *Shuang-huang-lian injection* was achieved from the second Chinese Medicine Factory of Harbin Pharm Group CO., Ltd. (Registered No. Z10960058).

### Animals and Handle

The protocol of the study was approved by the Ethics Committee of Beijing University of Chinese Medicine. A total of 96 male Sprague-Dawley rats (200±20 g) were commercially obtained from the institute of experimental animals in the *Weitonglihua* Laboratory Animal Technology Co., Ltd. (Beijing, China) (Rodent license No. SYXK 11-00-0039), housed in an environmentally controlled room at a constant temperature (23±2°C) and humidity (60±5%) on a 12 h light/dark cycle and provided with standard diet and water *ad libitum* for a one-week acclimation period. The rats’ rectal temperatures were measured two times per day using a digital thermometer for monitoring the regular rhythm of body temperatures at the last three days. The rats with a temperature difference that was greater than 0.5°C were excluded. Then rats were randomly divided into 3 groups: the normal control group (NC, *n* = 24), the pyretic model group (M, *n* = 24), and the model rats treated with *SHLI* group (*SHLI*, *n* = 24). Besides, the rest of the rats were taken as parallel control to detect exogenous compounds produced with *SHLI* metabolism.

The rats’ rectal temperatures were measured before modeling. The M and *SHLI* groups were subcutaneously injected with a 40% aqueous suspension of yeast (7.5 mL/kg) in the back below the nape of the rats (NC groups were given an equal volume of 0.9% saline). The rats in *SHLI* groups were then injected 600 mg/kg *SHLI* (dissolved in 0.9% saline) from the intraperitoneal (NC and M groups were given an equal volume of 0.9% saline instead). Six rats in each group were anesthetized with 10% chloral hydrate and blood samples containing heparin sodium anticoagulant were collected from the abdominal aortic at 5 hrs, 6 hrs, 8 hrs, and 10 hrs after *SHLI* or 0.9% saline was administrated.

### Preparation of Plasma Samples and UPLC-ESI-Q-TOF/MS Analysis

Plasma sample preparation was carried out by protein precipitation with methanol using Ostro 96-well plates with pressure valves (Ostro plates, Waters). Briefly, 400 µL of methanol was added to 100 µL plasma in an Ostro 96-well plate. The mixture was then quickly mixed by aspirating the samples 10 times using the micropipette and kept for 10 min. Vacuum was then applied to the Ostro plate to collect the mixture. Added 400 µL of methanol into the plate and repeated the steps once again. The prepared plasma samples were removed to the injection vial for UPLC-Q-TOF/MS analysis.

Metabolomics analysis was performed on an Waters Acquity UPLC system coupled to a Xevo G2-Q-TOF (Waters MS Technologies, Manchester, UK) equipped with an electrospray ionization source operating in positive and negative ion modes. An Acquity UPLC HSS T3 column (2.1×100 mm, 1.8 µm, UK) equipped with a binary solvent delivery system and an autosampler. The column was maintained at 45°C and eluted at a flow rate of 0.45 mL/min. The gradient mobile phase was a mixture of 0.1% formic acid in water (A) and 0.1% formic acid in acetonitrile (B). The proportion of mobile phase B was optimized as follows: 0∼10 min, 1∼99%, maintained 1 min with 99%, then returned to 1% at the next 1 min and held for 3 min for positive ion mode; 0∼5 min, 1∼99%, maintained 1 min with 99%, then returned to 1% at the next 1 min and held for 2 min for negative ion mode. The autosampler was maintained at 4°C and the injection volume was 5 µL. Data were collected from *m/z* 50 to *m/z* 1200. For positive ion mode, the capillary and cone voltage were set at 3 kV and 30 V. For negative ion mode, the capillary and cone voltage were set at 2.5 kV and 45 V. The desolvation gas was set at 800 L/h at a temperature of 450°C. The source temperature was set at 120°C. The cone gas was set at 15 L/h for positive ion mode and 30 L/h for negative ion mode. Leucine-enkephalin was used as the lock mass solution to ensure the accuracy and reproducibility.

### Data Processing and Multivariate Data Analysis

UPLC-Q-TOF/MS data of the plasma samples were processed using Waters Markerlynx XS software to transform the raw data into a single matrix containing aligned peaks with the *m/z*/retention time pair along with normalized peak intensities and a sample name. The matrix was then introduced to EZinfo 2.0 software for principal component analysis (PCA) and orthogonal partial least-squared discriminant analysis (OPLS-DA) analyses. All variables were Pareto scaled before analysis. In order to gain an overview of the rat plasma metabolic profiling; an unsupervised PCA method was used to give the comprehensive view of the clustering trends for the three groups. A supervised OPLS-DA analysis technique was used to search biomarkers based on the variable importance in the projection (*VIP >1*) between NC and M. The quality of these models can be explained byR^2^ and Q^2^ values. R^2^ indicates the goodness of fit displaying the variance explained in the model and Q^2^ indicates predictability displaying the variance in the data predictable by the model. The values of these parameters close to 1.0 indicating a reasonably good prediction for the constructed mathematical model.

### Identification of Plasma Biomarkers

For the identification of potential biomarkers, some available biochemical databases, such as HMDB (http://www.hmdb.ca/), KEGG (http://www.genome.jp/kegg/), METLIN (http://metlin.scripps.edu/), LIPIDMAPS (http://www.lipidmaps.org/) and Chemspider (http://www.chemspider.com) were used by comparing the accurate mass and fragments information obtained from UPLC-Q-TOF/MS.

### Comparative and Analysis of Plasma Metabolites

The statistical analysis of the relative intensity of biomarkers was performed by SPSS 17.0. The integration areas of the detected metabolites with high VIP values were first tested for the normality of the distribution. If the distribution followed the normality assumption, a parametric Student’s *t*-test was applied; otherwise, a nonparametric Mann-Whitney U test was performed to detect statistically significant metabolites that were increased or decreased between groups. Differences were considered significant at a value of *P<0.05*. Statistic analysis of One-way ANOVA was also performed for the rectal temperature results.

Metabolomic pathway analysis was performed with MetPA (metabolomics pathway analysis) based on potential metabolite biomarkers. MetPA is a user-friendly, web-based tool for pathway analysis and visualization of metabolomic data within the biological context of metabolic pathways [49]. For the pathway analysis algorithms, Hypergeometric Test was used for over representation analysis, and Relative-betweeness Centrality was used for pathway topology analysis.

## Supporting Information

Table S1 **Drug-induced components and their metabolites in the plasma of Shuang-huang-lian injection (** ***SHLI)*** **treated rats.**
(DOC)

Table S2 **Summary of pathway analysis with MetPA.**
(DOCX)
