# Supplementary material for: Plasma Metabolomic Profiling to Reveal Antipyretic Mechanism of Shuang-Huang-Lian Injection on Yeast-Induced Pyrexia Rats
Source: PLoS One. 2014 Jun 18;9(6):e100017. doi: 10.1371/journal.pone.0100017 (PMC4062457; doi:10.1371/journal.pone.0100017)
Supplement: Table S1 — Drug-induced components and their metabolites in the plasma of Shuang-huang-lian injection ( SHLI) treated rats. (DOC) [file pone.0100017.s001.doc]

Table S1 Drug-induced components and their metabolites in the plasma of Shuang-huang-lian injection (SHLI) treated rats.

| **No.** | **t_R_(min)** | ***m/z*** | **Metabolites** | **Quasi-molecular ion** | **Formula** | **Proposed structure** | **MS/MS** | **Loss** |
| --- | --- | --- | --- | --- | --- | --- | --- | --- |
| 1 | 3.8353 | 447.0921 | Baicalein 7-O-glucuronide^a^ | [M+H]^+^ | C_21_H_18_O_11_ |  | 271.0607 | -C_6_H_8_O_6_ |
| 2 | 3.8365 | 271.0604 | Baicalein^a^ | [M+H]^+^ | C_15_H_10_O_5_ |  | 253.0495  123.0083 | -H_2_O  -C_9_H_8_O_2_ |
| 3 | 3.3901 | 623.1240 | Baicalein 7,6-diglucuronide^a^ | [M+H]^+^ | C_27_H_26_O_17_ |  | 447.0913  271.0607 | -C_6_H_8_O_6_  -C_12_H_16_O_12_ |
| 4 | 4.3163 | 447.0922 | Baicalein 6-O-glucuronide | [M+H]^+^ | C_21_H_18_O_11_ |  | 271.0614  123.0083 | -C_6_H_8_O_6_  -C_15_H_16_O_8_ |
| 5 | 5.6489 | 285.0761 | 7,5-Dihydroxy-6-methoxyflavone^a^ | [M+H]^+^ | C_16_H_12_O_5_ |  | 270.0534  253.0468  225.0565 | -CH_3_  -CH_4_O  -C_2_H_4_O_2_ |
| 6 | 5.2247 | 231.0654 | 4-(3-Hydroxy-1H-inden-2-yl)-2-oxo-3-butenoic acid | [M+H]^+^ | C_13_H_10_O_4_ |  | 213.0533  118.0870 | -H_2_O  -C_4_HO_4_ |
| 7 | 2.2408 | 151.0395 | 5-Hydroxy-2-coumaranone | [M+H]^+^ | C_8_H_6_O_3_ |  | 133.0655 | -H_2_O |
| 8 | 2.2249 | 211.0609 | 5-Hydroxyferulic acid methyl ester | [M+H]^+^ | C_10_H_10_O_5_ |  | 193.0497  175.0394  133.0311 | -H_2_O  -H_4_O_2_  -CH_4_O_3_ |
| 9 | 2.4655 | 445.0771 | Baicalein 7-O-glucuronide^a^ | [M-H]^-^ | C_21_H_18_O_11_ |  | 269.0448  175.0252  113.0241 | -C_6_H_8_O_6_  -C_15_H_10_O_5_  -C_16_H_12_O_8_ |
| 10 | 2.2044 | 621.1083 | Baicalein 7,6-diglucuronide | [M-H]^-^ | C_27_H_26_O_17_ |  | 445.0770  269.0441 | -C_6_H_8_O_6_  -C_12_H_16_O_12_ |
| 1811 | 2.7288 | 445.0770 | Baicalein 6-O-glucuronide | [M-H]^-^ | C_21_H_18_O_11_ |  | 269.0460 | - C_6_H_8_O_6_ |
| 12 | 1.6652 | 353.0873 | Chlorogenic acid^a^ | [M-H]^-^ | C_16_H_18_O_9_ |  | 191.0563 | -C_9_H_6_O_3_ |
| 13 | 2.6619 | 459.0924 | Baicalein 6-methyl ether 7-glucuronide | [M-H]^-^ | C_22_H_20_O_11_ |  | 283.0599  268.0340 | - C_6_H_8_O_6_  -C_7_H_10_O_6_ |
| 14 | 1.6713 | 373.1132 | Geniposidic acid | [M-H]^-^ | C_16_H_22_O_10_ |  | 191.0552  173.0449  179.0346 | -C_7_H_11_O_6_  -C_9_H_12_O_5_  -C_7_H_14_O_6_ |
| 15 | 1.9016 | 403.1238 | Gardenoside | [M-H]^-^ | C_17_H_24_O_11_ |  | 179.0540  165.0546  149.0579 | -C_11_H_12_O_5_  -C_8_H_14_O_8_  -C_8_H_14_O_9_ |
| 16 | 1.6017 | 389.1083 | Monotropein | [M-H]^-^ | C_16_H_22_O_11_ |  | 345.1227  183.0648  165.0553 | -CO_2_  -C_7_H_10_O_7_  -C_7_H_12_O_8_ |

^a^Metabolites were identified based on database information in METLIN.
