# Supplementary material for: Plasma Metabolomic Profiling to Reveal Antipyretic Mechanism of Shuang-Huang-Lian Injection on Yeast-Induced Pyrexia Rats
Source: PLoS One. 2014 Jun 18;9(6):e100017. doi: 10.1371/journal.pone.0100017 (PMC4062457; doi:10.1371/journal.pone.0100017)
Supplement: Table S2 — Summary of pathway analysis with MetPA. (DOCX) [file pone.0100017.s002.docx]

Table S2. Summary of pathway analysis with MetPA. (1) Valine, leucine and isoleucine biosynthesis. (2) Glycerophospholipid metabolism. (3) Synthesis and degradation of ketone bodies. (4) Riboavin metabolism. (5) Butanoate metabolism. (6) Valine, leucine and isoleucine degradation. (7) Aminoacyl-tRNA biosynthesis.

|  | Total | Expected | Hits | Raw p | Holm p | FDR | Impact |
| --- | --- | --- | --- | --- | --- | --- | --- |

| Valine, leucine and isoleucine biosynthesis | 11 | 0.06 | 1 | 0.061 | 1.0 | 1.0 | 0.33 |
| --- | --- | --- | --- | --- | --- | --- | --- |
| Glycerophospholipid metabolism | 30 | 0.17 | 1 | 0.159 | 1.0 | 1.0 | 0.04 |
| Synthesis and degradation of ketone bodies | 5 | 0.03 | 1 | 0.028 | 1.0 | 1.0 | 0.0 |
| Riboavin metabolism | 11 | 0.06 | 1 | 0.061 | 1.0 | 1.0 | 0.0 |
| Butanoate metabolism | 20 | 0.11 | 1 | 0.109 | 1.0 | 1.0 | 0.0 |
| Valine, leucine and isoleucine degradation | 38 | 0.22 | 1 | 0.198 | 1.0 | 1.0 | 0.0 |

| Aminoacyl-tRNA biosynthesis | 67 | 0.38 | 1 | 0.325 | 1.0 | 1.0 | 0.0 |
| --- | --- | --- | --- | --- | --- | --- | --- |
